# Can nutrition interventions tackle the global insulin affordability via improving diabetes management and reducing insulin demand?

**DOI:** 10.1186/s41256-022-00247-1

**Published:** 2022-05-11

**Authors:** Victoria Lu, Jiandi Zhang, Guoxun Chen

**Affiliations:** 1 Yantai Zestern Biotechnique Co. LTD, Yantai, Shandong 264670 China; 2 Department of Nutrition, University of Tennessee at Knoxville, 229 Jessie Harris Building, 1215 West Cumberland Avenue, Knoxville, TN 37909 USA

**Keywords:** Diabetes, Food insecurity, Global health, Insulin affordability, Insulin price, Nutrition intervention

## Abstract

Diabetes, a global health concern, requires insulin therapy. As insulin demand and prices rise dramatically, insulin affordability has increasingly become an issue facing patients with diabetes worldwide. To cut insulin costs, many patients ration their supply, which may have dire health consequences. This particularly affects lower-income populations, who are often forced to choose between purchasing their medications or paying for other necessities. Nutrition might be one solution for this. This commentary aims to provide comprehensive insight with historical context into intersectional components of diabetes in the global arena through analyses of insulin affordability, coupled with the critical role of nutrition intervention after searching the PubMed for relevant articles. More studies in personalized nutrition, supplementations, and dietary behaviors may develop evidence-based nutrition interventions to control diabetes. We argue that alongside price regulation, a greater focus to nutrition to address issues of food insecurity and food assistance programs may help to improve insulin affordability.

## Background

The affordability of insulin to manage diabetes has evolved into a global health issue, which is especially taunting 100 years after its discovery [1, 2]. According to the statistics by IDF Diabetes Atlas, the adults with diabetes had reached 537 million globally in 2021. For the past century, dramatic changes from medicine to agriculture and food sciences have provided solutions along with challenges for diabetes management. The recombinant DNA technologies allow the massive production of recombinant human insulin and its analogues. Conversely, less nutrient-rich foods facilitate the development of chronic metabolic diseases. This augments the demand for more insulin to control diabetes. After Frederick Banting and his colleagues discovered insulin and expressed firmly that insulin is a life-saving medication, this great gift to humanity, however, now embodies an adverse reality characterized by skyrocketing insulin prices. This commentary is to discuss the current dilemmas of diabetes management in a global health perspective. In December 2021 and March 2022, we searched PubMed using key words “insulin affordability” and “nutrition” to retrieve relevant articles for this commentary.

## A brief history of diabetes and insulin therapy

The symptoms of diabetes have been observed for thousands of years in ancient Egypt, Indian, and China. The link between diabetes and pancreas was observed in 1890. Insulin as a pancreatic secretion was successfully extracted and purified by Fredrick Banting and his colleagues in 1921, a work that won the Nobel Prize in 1923. After its discovery, insulin was extracted from animal pancreas for clinical uses. The clinical use of insulin led to the discovery of two types of diabetes, type 1 diabetes (T1D) and type 2 diabetes (T2D). The improvement of life expectancy in T1D patients after insulin therapy demonstrates its essentiality. The introduction of recombinant human insulin in 1982 enables more patients to be treated. Later, rapid-acting, long-acting insulin analogues, inhaled and biosimilar insulin were introduced in 1996, 2002, 2006 and 2021, respectively. Now, the available forms of insulin include extractions from animal pancreas, recombinant human insulin, insulin analogues, inhaled human insulin and biosimilar insulin [3]. Although the market share of analogue insulin increased from 1999 to 2009, the human recombinant insulin remains dominant in the middle and low-income countries [1, 2].

Before the insulin therapy, diabetes was treated with starvation or dietary restriction. For T2D patients, lifestyle changes have been the first choice before any drug and insulin therapies. As insulin is used for both T1D and T2D, its demand along with other healthcare spendings for diabetes increases, which poses a prodigious challenge worldwide [1, 2].

## The insulin affordability challenge

The insulin affordability is a problem not only for low-income countries, but also for the developed ones like the United States (U.S.), which has higher prices than other developed countries such as United Kingdom. Three large companies, Novo Nordisk, Eli Lilly, and Sanofi, control 99% of the global insulin market in terms of value and 96% in terms of volume [1, 2]. The affordability of insulin and other chemical drugs for diabetes treatment varies among different countries. The average list price of 4 types of insulin increased by 15% annually from 2012 to 2016, whereas their net prices from producers only increased by 36% and 3% in the same period, respectively. From 2002 to 2012, the expenditure of insulin for diabetes treatment in adults in the U.S. increased from $2.6 to 15.4 billion.

The reasons of high insulin prices include the presence of patients who need insulin to survive, monopoly, purposed strategies of incremental patents (evergreening practice), barriers to biosimilar entry (not considered generics), middlemen controlling the market share, and lobbying power to maintain the status quo [4]. As the recombinant human insulin has been commercialized for about 40 years, the development and innovation are not the major costs. Generic-drug makers may help to reduce the prices when they start to produce biosimilar insulin products. To address the insulin price issue, the U.S. 116th Congress (2019–2020) introduced seven bipartisan bills in the following five categories: improving price transparency, limiting out-of-pocket costs, changing biosimilar regulations, certifying prices, and permitting importation [5].

## A conceptual framework of nutrition and insulin affordability interactions

T2D can be managed via healthy diets, physical activity and supplementations such as zinc [6]. However, food insecurity, defined as limited or uncertain availability of nutritionally adequate and safe foods, contributes to risk factors of T2D, and management of diabetes [7]. Subjects experiencing food insecurity may purchase more calorie-dense foods that are cheaper but richer in carbohydrates and saturated fats than healthier alternatives due to financial and low socioeconomic reasons, which is correlated with lower dietary quality and poorer glycemic control over time.

About half of the patients with hyperglycemia in an inner-city emergency department experienced food insecurity, with approximately one-third of those who had used less than the intended amount of insulin previously, presenting the interaction between food insecurity and insulin usage. Referrals to food resources and nutrition counseling have been suggested as a method to assist diabetic individuals experiencing food insecurity. The diets of Americans with low socioeconomic status contain excessive refined carbohydrates, sodium, saturated fats, and added sugar, which are the nutritional issues faced by the Supplemental Nutrition Assistance Program (SNAP) of the US Department of Agriculture [8].

**Fig. 1:**
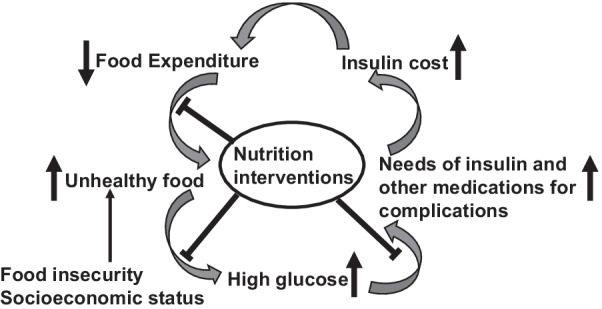
A conceptual role of nutrition interventions in the improvement of insulin affordability

As depicted in Fig. 1, we propose a conceptual framework to show that a vicious cycle due to food insecurity and management of diabetes appears to exist. This might have contributed to increased insulin demand and its price drive-up. Here, individuals with diabetes and lower socioeconomic status often experience food insecurity, propelling choices to consume unhealthy foods such as those containing excessive refined carbohydrates, sodium, saturated fats, and added sugar. This leads to the uncontrolled rise of blood glucose, which requires more insulin and other medications to manage. When insulin and other medications prices rise, less budget is available for healthy foods, which in turn drives the consumption of more unhealthy foods that further increases the blood glucose to a level needing additional insulin to control. Eventually, the affordability of insulin becomes an even more magnified issue.

Nutrition interventions may reduce the risk of diabetes, improve the glycemic management, and reduce the needs of insulin, which may indirectly improve the insulin affordability. Data collected from clinical trials, meta-analysis of micronutrient supplemental studies, prospective observational studies about dietary patterns, and personalized or precision nutrition can provide evidence-based intervention strategies to help patients control blood glucose and reduce the needs of insulin and other medications. Nutrition interventions should contribute to tackle the insulin affordability challenge in the following ways.

1. Adequate education of nutrition and diabetes will help people with diabetes and lower socioeconomic status to choose and consume healthy foods. After summarizing the technologies used to help T1D patients, the ADS/ADEA/APEG/ADIPS Working Group in Australia has recommended that focus of diabetes treatment should shift from strict trial-based glycemic criteria to personalized approaches to consider the broad spectrum of benefits obtained by these technologies [9]. Personalized or precision nutrition studies have shown some promise to control glycemia [10]. The benefits may have long-term impacts on the health of the individuals and populations as the nutrition exposure has memories in the form of epigenetics. The educated patients may choose and control their dietary intakes correctly, manage the disease accurately, and in turn lower the needs of insulin.
2. Nutrition community and clinicians can collaborate with policy makers to incentivize choices of healthy foods and limit the access of unhealthy foods through imposing stricter guidelines and legislation. SNAP is evidently an ideal tool that would benefit from engineering a capability to use financial incentives to facilitate healthy food choices and improved eating behaviors of those with low income and socioeconomic status to favorably reduce diabetes prevalence. This certainly will reduce all chronic metabolic disease prevalence affected by poor dietary choices.
3. Scientists can conduct well designed research to enable advanced technologies capable of aiding patients to manage blood glucose though personalized nutrition. As insulin is administrated via injection or pumps, affordability of other accessory healthcare items for diabetes such as strips of glucometer or continuous blood monitoring systems also need to be considered [1]. Clearly, with the efforts of all stake holders including the World Health Organization, the United Nations, government agencies in those countries, researchers and clinicians, insulin will become affordable to every patient with diabetes.

## Conclusions

When technologies for insulin delivery and glucose sensing become more reliable and accurate, more nutrition intervention practices are anticipated. They will not only demonstrate the power of nutrition interventions, but also drive down the insulin demands, and in turn, increase its availability and affordability. As insulin affordability has gradually become a global health issue, professional organizations, government agencies, insulin manufacturers, clinicians, and nutritionists can act cohesively and coordinately to tackle this challenge.

## Data Availability

Not applicable.

## Abbreviations

SNAP: Supplemental Nutrition Assistance Program
T1D: Type 1 diabetes
T2D: Type 2 diabetes
US: The United States
